# A new Mfn-2 related synthetic peptide promotes vascular smooth muscle cell apoptosis via regulating the mitochondrial apoptotic pathway by inhibiting Akt signaling

**DOI:** 10.1186/s12967-021-03064-1

**Published:** 2021-09-19

**Authors:** Xinxin Zhang, Xiangyu Xu, Li Lu, Xiaoning Wan, Yating Qin, Weibin Ruan, Chao Lv, Lin He, Xiaomei Guo

**Affiliations:** 1 Department of Cardiology, Tongji Hospital, Tongji Medical College, Huazhong University of Science and Technology, Wuhan, 430030 China; 2 Department of Cardiology, The Second Hospital, Cheeloo College of Medicine, Shandong University, Shandong, China; 3 Department of Cardiology, Renmin Hospital of Wuhan University, Wuhan, China; 4 Bio-X Institutes, Key Laboratory for the Genetics of Developmental and Neuropsychiatric Disorders (Ministry of Education), Shanghai Jiaotong University, Shanghai, China

**Keywords:** MRSP, Neointimal hyperplasia, Apoptosis, PI3K/Akt signaling

## Abstract

**Background:**

Restenosis after angioplasty is a major challenge for the treatment of coronary artery diseases. Facilitation of vascular smooth muscle cell (VSMC) apoptosis may be an attractive approach to decrease the incidence of restenosis. We synthesized a 16-amino acid mitofusin-2 (Mfn-2) gene related peptide (MRSP) based on the sequence of the p21^ras^ signature motif, the smallest functional sequence of the Mfn-2 gene with proapoptotic properties in VSMC. We investigated whether MRSP enhanced apoptotic activities to inhibit VSMC accumulation and neointimal hyperplasia in rats with carotid balloon injury.

**Methods:**

VSMCs were treated with different concentrations of MRSP, the PI3K agonist 740 Y-P and the inhibitor LY294002. Cell apoptosis and related pathway molecules were assessed. MRSP was also given to rats with carotid artery balloon injury. Neointimal hyperplasia and cell apoptotic pathways were detected.

**Results:**

In vitro experiments revealed that MRSP treatment significantly increased VSMC apoptosis and induced increases in procaspase-9 cleavage, caspase-3 activation, cytochrome c release from mitochondria to the cytoplasm and the Bax/Bcl-2 ratio but not caspase-8 expression, indicating that the mitochondrial apoptotic cascade was activated by MRSP, which might be attributed to suppression of the PI3K/Akt signaling pathway. We further found that the PI3K agonist 740 Y-P prevented and that the inhibitor LY294002 strengthened the proapoptotic effects of MRSP. MRSP strongly inhibited neointimal hyperplasia and VSMC accumulation, but increased VSMC apoptosis in the vascular wall after balloon injury. Moreover, MRSP substantially enhanced Bax and cleaved caspase-3 expression and decreased Bcl-2 levels in intima, accompanied by decreased levels of phosphorylated Akt and PI3K in vivo.

**Conclusions:**

Taken together, the present study showed that MRSP treatment results in a strong proapoptotic effect by activating the mitochondrial apoptotic cascade through suppression of the PI3K/Akt pathway.

**Supplementary Information:**

The online version contains supplementary material available at 10.1186/s12967-021-03064-1.

## Induction

Atherosclerotic diseases have become the number one cause of mortality and are a threat to human health, with increased morbidity and a younger trend [1]. Percutaneous coronary intervention (PCI) has been used extensively in the treatment of severe coronary atherosclerotic diseases [2]. However, there remain concerns about the long-term prognosis of patients suffering from postoperative restenosis (RS) [3–5], which is caused by neointimal hyperplasia [6] in the area of stent angioplasty. Vascular smooth muscle cells (VSMCs) are the major constituents of hyperplastic neointima. After the vascular endothelium is damaged, VSMCs undergo a phenotypic transformation and show aberrant proliferation with insufficient apoptosis, migration to the intima and secretion of extracellular matrix and multiple cytokines, thus leading to neointimal hyperplasia [6, 7]. Thus, approaches antagonizing the progression of these pathogenic processes are likely to be effective in inhibiting the development of RS, especially increased apoptosis. For instance, activation of Fas ligand could increase VSMC apoptosis and attenuate neointimal hyperplasia in the carotid artery of rats with balloon injury [8]. Recent studies have shown that VSMCs in the intimal proliferative stage are resistant to apoptosis and that mitigation of such resistance is highly effective in accelerating VSMC apoptosis and decreasing neointimal thickening [9]. Consequently, there remains a need for novel treatments targeting VSMC apoptosis and RS.

Mitofusin 2 (Mfn-2), located on the outer mitochondrial membrane, is important in regulating outer mitochondrial membrane fusion and maintaining mitochondrial morphology [10, 11]. Our previous studies have demonstrated that Mfn-2 acts as an endogenous Ras inhibitor and has critical roles in cell apoptosis and vascular proliferative disorders [12, 13]. Further studies demonstrated that adenoviral gene transfer of rMfn-2 significantly increases VSMC apoptosis in balloon-injured rat carotid arteries and alleviates angioplasty-induced neointima formation [12, 13], independent of its function in mitochondrial fusion. Importantly, overexpression of Mfn-2 significantly drives apoptotic death in multiple cell lines, implying that the Mfn-2 gene is important determinant of cell fate [14, 15]. Along with its antiproliferative effect, the proapoptotic function of Mfn-2 suggests it as a potential therapeutic target in treating RS after stenting.

The Mfn-2 gene encodes 757 amino acids, which is not conducive to further clinical research. Based on the effects of different structural sequences of Mfn-2 on VSMCs, we discovered that the p21ras signature motif, encoding 16 amino acids, was the minimum functional sequence possessing antiproliferative and proapoptotic effects. Then, we artificially synthesized the Mfn-2 gene-related peptide (MRSP) conjugated to the HIV-1TAT protein transduction domain, which has been used to transduce macromolecules into cells in vitro and in vivo. In the present study, we focused on the apoptotic effect of MRSP on VSMCs and the effect on neointimal hyperplasia in rats with carotid balloon injury and explored relevant potential mechanisms, to provide a theoretical basis for the drug treatment of RS.

## Materials and methods

### Cell culture

VSMCs from the thoracic aorta of Wistar Kyoto (WKY) rats were kindly provided by Professor KH Chen at the National Institutes of Health (Bethesda, USA). Cells from passages 3 to 9 were used throughout this study. Cells were cultured in Dulbecco's modified Eagle's medium (DMEM, Gibco, Grand island, NY, USA) containing 10% fetal bovine serum (FBS, Gibco, Grand island, NY, USA) and 1% penicillin/streptomycin (Gibco, Grand island, NY, USA) at 37 ℃ in 5% CO_2_. The culture medium was changed every 2 to 3 days.

### Fluorescence colocalization detection

VSMCs were plated on cover slips in 6-well plates at 2 × 10^5^ cells/well, serum starved for 24 h and then treated with MRSP-His at various time points (6 h, 12 h, 18 h, 24 h) at 37 ℃ with or without 10% FBS. Cover slips were processed for immunofluorescence with MitoTracker CMXRos (Invitrogen, Carlsbad, CA) and MRSP, and colocalization analysis, as previously described. First, viable cells were stained with MitoTracker CMXRos, followed by fixed and rupture of cell membranes. Then cells were blocked and probed with primary antibody overnight at 4 ℃. The antibody used was anti-His tag antibody (AE003, Abclonal, Boston, USA), and the secondary antibody was Alexa-Fluor 488 (green, Servicebio Biotechnology, Wuhan, China). Then the coverslips were stained with DAPI (Servicebio Biotechnology, Wuhan, China) to stain the nuclei. Images were obtained on a fluorescence microscope (Olympus, Japan), and a colocalization analysis was conducted by Image J.

### Apoptosis assay

Cells were seeded in 6-well plates at 2 × 10^5^ cells/well and synchronized by serum deprivation for 24 h. Then, VSMCs were incubated with different concentrations (25 and 50 μM) of MRSP over a time series (12 and 24 h) with or without 10% FBS. The pro-apoptotic effects in different groups were demonstrated by Annexin V-FITC/PI flow cytometric assays. The assay was carried out according to the manufacturer's instructions (Beyotime, Beijing, China). Briefly, the treated VSMCs were harvested with trypsin, washed in PBS, centrifuged at 2000*g* for 5 min and resuspended in binding buffer at a concentration of 10^6^ cells per 1 ml, followed by incubation with 5 μl of Annexin V-FITC for 15 min and subsequent incubation with 5 μl of PI solution for 5 min at room temperature in the dark. The cell suspension was analyzed within 30 min and at least 10,000 stained cells of each sample were analyzed by flow cytometry. The apoptosis ratio was determined as the sum of early and late apoptotic rates. The experiments were carried out in triplicates.

### Measurement of DNA fragmentation by cell death detection ELISA kit

VSMCs were treated as described above. The apoptotic response was measured by the Cell Death Detection ELISA kit according to the manufacturer's instructions (Roche Applied Science, USA). Briefly, VSMCs (1 × 10^4^ cells/well) in 96-well microtiter plates were incubated with different concentrations of MRSP for various periods of time, and the absorbance was examined at 405 nm. The experiments were carried out in triplicates.

### TUNEL assay

VSMCs were treated as described above, fixed in 4% paraformaldehyde solution, washed three times with PBS, incubated for 5 min with 5% Triton-X 100, washed three times with PBS, and then labeled with the DeadEnd Colorimetric TUNEL System (Beyotime, Beijing, China) one hour at 37 ℃, followed by incubating with DAPI for 5 min. Images were captured by a fluorescence microscope. Apoptosis of the neointima was determined following the manufacturer's instructions. In brief, 5 μm sections were deparaffinized, rehydrated and incubated with proteinase K, followed by incubation with fresh 3% hydrogen peroxide and then incubation with TUNEL reaction mixture for 60 min at 37 ℃. After blocking, the anti-fluorescein antibody was applied to the sections for 30 min at 37 ℃ in the dark. The apoptosis ratio was represented by the TUNEL-positive rate, which was equal to the number of TUNEL-positive cells divided by the total cell number * 100%. The experiments were carried out in triplicates.

### Cytochrome c release

Firstly, Cells were seeded in 6-well plates at 2 × 10^5^ cells/well and synchronized by serum deprivation for 24 h. Then, VSMCs were incubated with HIV1TAT or nothing for 12 h, which detected the difference between the group of HIV1TAT alone and the control group. Then VSMCs were treated as described above. For determination of the effect of MRSP on cytochrome c release, mitochondrial and cytosolic proteins were separated using the Cell Mitochondria Isolation Kit (Beyotime, Beijing, China). The expression level of cytochrome c was analyzed by western blots. The expression of cytochrome c oxidase 4 (COX4, A11631, ABclonal, Boston, USA), a mitochondrial marker was monitored to determine the purity of the cytosol and mitochondrial components. The experiments were carried out in triplicates.

### Western blot assay

VSMCs were treated as described above. Protein levels were measured by western blot analysis as described previously. Cell or tissue proteins were extracted using RIPA buffer (Boster Biological Technology, Wuhan, China) with a protease inhibitor mixture, and protein concentrations were determined using a bicinchoninic acid (BCA) kit (Boster Biological Technology, Wuhan, China). Equal amounts of proteins (20 ug) were loaded, separated by SDS-PAGE, transferred onto polyvinylidene difluoride (PVDF) membranes and blocked with 5% BSA in TBST for 2 h at room temperature. Then, the membranes were washed and incubated overnight with primary antibodies. The primary antibodies included Mfn-2 (1:1000, #9482, CST, Boston, USA), PI3K (1: 1000, #4249, CST, Boston, USA), phospho-PI3K (1: 1000, #4228, CST, Boston, USA), Akt (1: 1000, #4691, CST, Boston, USA), phospho-Akt (1: 1000, #4060, CST, Boston, USA), ERK1/2 (1:1000, #4695, CST, Boston, USA), p-ERK1/2 (1:1000, #4370, CST, Boston, USA), p38 (1:1000, #9212, CST, Boston, USA), p-p38 (1:1000, #9211, CST, Boston, USA), JNK (1:1000, #9252, CST, Boston, USA), p-JNK (1:1000, #9255, CST, Boston, USA), Bcl2 (1: 1000, A0208, ABclonal, Boston, USA), Bax (1: 1000, #2772, CST, Boston, USA), cleaved caspase-3 (1: 1000, #9664, CST, Boston, USA), cleaved caspase-8 (1: 1000, 13423-1-AP, Proteintech, Chicago, IL, USA), cleaved caspase-9 (1: 1000, #9507, CST, Boston, USA), cytochrome c (1:1000, A4912, ABclonal, Boston, USA) and β-actin (1:10,000, AC004, ABclonal, Boston, USA). After that, the membranes were incubated with peroxidase-conjugated secondary antibodies, and ECL (Boster Biological Technology, Wuhan, China) and then visualized by autoradiography.

### Rat carotid artery balloon denudation injury

All animal procedures were in compliance with the National Institutes of Health Guide for the Care and Use of Laboratory Animals under approval by the Institutional Animal Care and Use Committee of Tongji Medical College, Huazhong University Science and Technology, Wuhan, China.

Male Sprague–Dawley rats (280–320 g, Institutional Animal Research Committee of Tongji Medical College, Wuhan, China) were used. Animals were housed in a room at a controlled temperature of 23 ℃ with a 12:12-h dark–light cycle. The rats had free access to water and rodent chow diet throughout the experiment. Animals were randomly divided into three groups (n = 12 each group): sham, model, and MRSP (10 mg/rat). The animals were intraperitoneally anesthetized with ketamine hydrochloride (70 mg/kg) and xylazine (5 mg/kg) and heparinized with 100 U/kg heparin sodium through the tail vein. The 2F Fogary balloon embolectomy catheter (Baxter Healthcare Corp, IL, USA) was inserted through an incision in the left external carotid artery into the origin of the common carotid artery. The balloon was distended, and the procedure was repeated 3 times with rotation to produce a substantial vascular injury. Immediately following balloon injury, carotid artery surface was painted with 200 μl of 25% Pluronic F-127 gel solution (Sigma-Aldrich, St Louis, MO, USA), a thermosensitive amphiphilic polymer, containing MRSP or nothing. The control rats that underwent sham surgery were treated as described above without balloon-injury. The left external carotid artery was ligated after removal of the catheter, and the wound was closed. All groups were maintained for 1 or 2 weeks on a normal chow diet after surgery.

One week and two weeks after balloon injury, the rats were euthanized and carotid arteries were acquired for further exploration.

### Histomorphometric analysis

Samples were fixed in 4% paraformaldehyde for 24 h, embedded in paraffin and sectioned into 4 μm thick slices. Then routine hematoxylin and eosin staining (HE, Beyotime, Beijing, China) was performed. Cross-sectional areas of the lumen, neointima, and media were observed by HE staining, and morphometric analysis was carried out using image analysis software. The intima-to-media ratio (I/M ratio), which represents neointimal proliferation, was then calculated.

### Immunohistochemical staining

After carotid artery slide deparaffinization and rehydration, endogenous peroxidase deactivation was achieved by 3% H_2_O_2_ for 10 min, followed by washing with PBS and antigen retrieval. Then antigen blocking serum was applied for 30 min, followed by overnight incubation at 4℃ with a primary anti-Bax (1:200) antibody, anti-Bcl (1:200) antibody, and anti-cleaved-caspase3 (1:200) antibody. After that, these slides were incubated with the biotinylated secondary antibody for 60 min at room temperature. Subsequently, 3′3-diaminobenzidine solution (DAB kit) was used for visualization. For quantitative analysis of the results of immunohistochemical staining, the mean optical density value in each region was calculated as: IOD/total area.

### Statistical analysis

All data are expressed as means ± SD. The error bar is generally the results of three independent experiments under one condition and is represented by SD. A One-way analysis of variance (ANOVA) followed by the Boneferroni procedure was performed to determine the statistical significance. The comparison of two groups was tested by Student's unpaired *t* test. Differences with p < 0.05 were considered statistically significant.

## Results

### Artificial synthesis of MRSP and its sorting in VSMCs

**Fig. 1:**
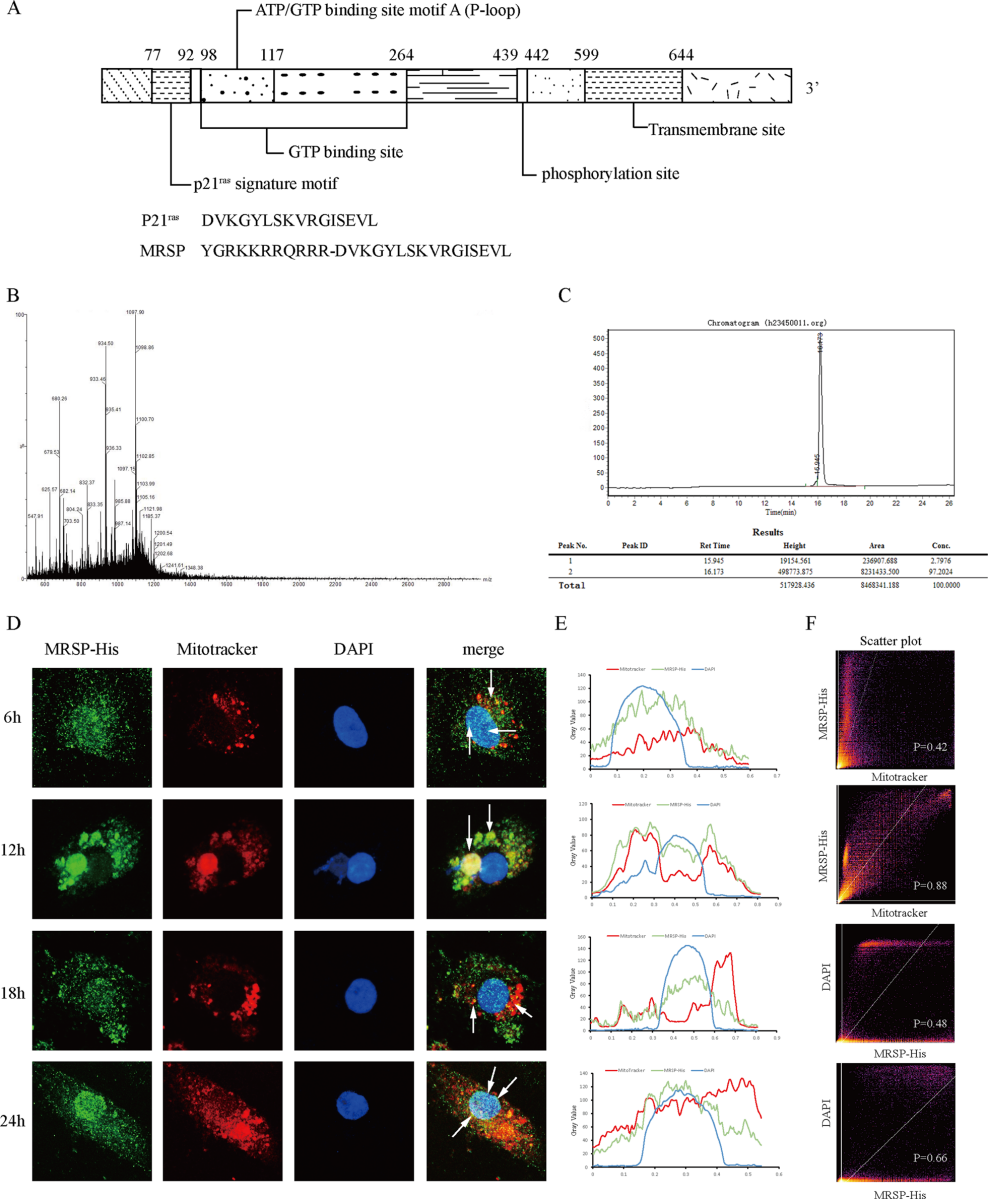
The basic situation of MRSP and its sorting at different time points. **A** The multiple structural elements of Mfn-2 and the amino acid sequence of the p21^ras^ signature motif and MRSP. **B** and **C** It was identified by mass spectrometry and chromatography. **D** The sorting of MRSP at the mitochondria and cell nucleus at different time points. **E** The level of overlay of MRSP-His, Mitotracker and DAPI. **F** Colocalization analysis with Pearson’s correlation coefficient of (**D**). n = 3 for this experiment

Using computer software to analyze the protein structure and function, we identified the structural elements of this gene and the amino acid sequence of the p21^ras^ signature motif, namely, DVKGYLSKVRGISEVL (Fig. 1A). Then, we artificially synthesized the peptide MRSP composed of p21^ras^ signature motif conjugated to the HIV-1 TAT protein transduction domain, i.e. YGRKKRRQRRR at the C-terminal. In addition, the sample was identified by mass spectrometry (Fig. 1B) and chromatography (Fig. 1C) as a white lyophilized powder with a molecular weight of 3305 and a purity of 97.2%.

To investigate further sorting of MRSP, we employed fluorescence colocalization analysis to test whether MRSP colocalized with MitoTracker Red dye and with DAPI. First, we artificially added His-tagged protein to MRSP to facilitate visualization. After MRSP-His intervention, there possibly was colocalization with MitoTracker and DAPI at the 6 h time point (Fig. 1D–F, Pearson’s correlation coefficient, P = 0.42 and 0.62 for MitoTracker and DAPI, respectively). Figure 1D revealed that the overlap between the green fluorescence signals of MRSP-His and the red fluorescence signals of mitochondria was significantly higher at the 12 h time point (Pearson’s correlation coefficient, P = 0.88). This result indicated that MRSP-His had high colocalization with mitochondria. As shown in Fig. 1D–F, MRSP-His showed lower colocalization with DAPI after 18 h incubation (Pearson’s correlation coefficient, P = 0.48), which was further increased by 24 h (Pearson’s correlation coefficient, P = 0.66) along with the low colocalization with mitochondria at the same time (Fig. 1D). Collectively, these data indicated that MRSP may be significantly internalized from the extracellular fluid and tended to sort from early mitochondria into the cell nucleus. Next, we will perform the colocalization with other organelles, and explore the interaction and detailed mechanisms.

### MRSP changed the level of endogenous Mfn-2 and promoted VSMC apoptosis

**Fig. 2:**
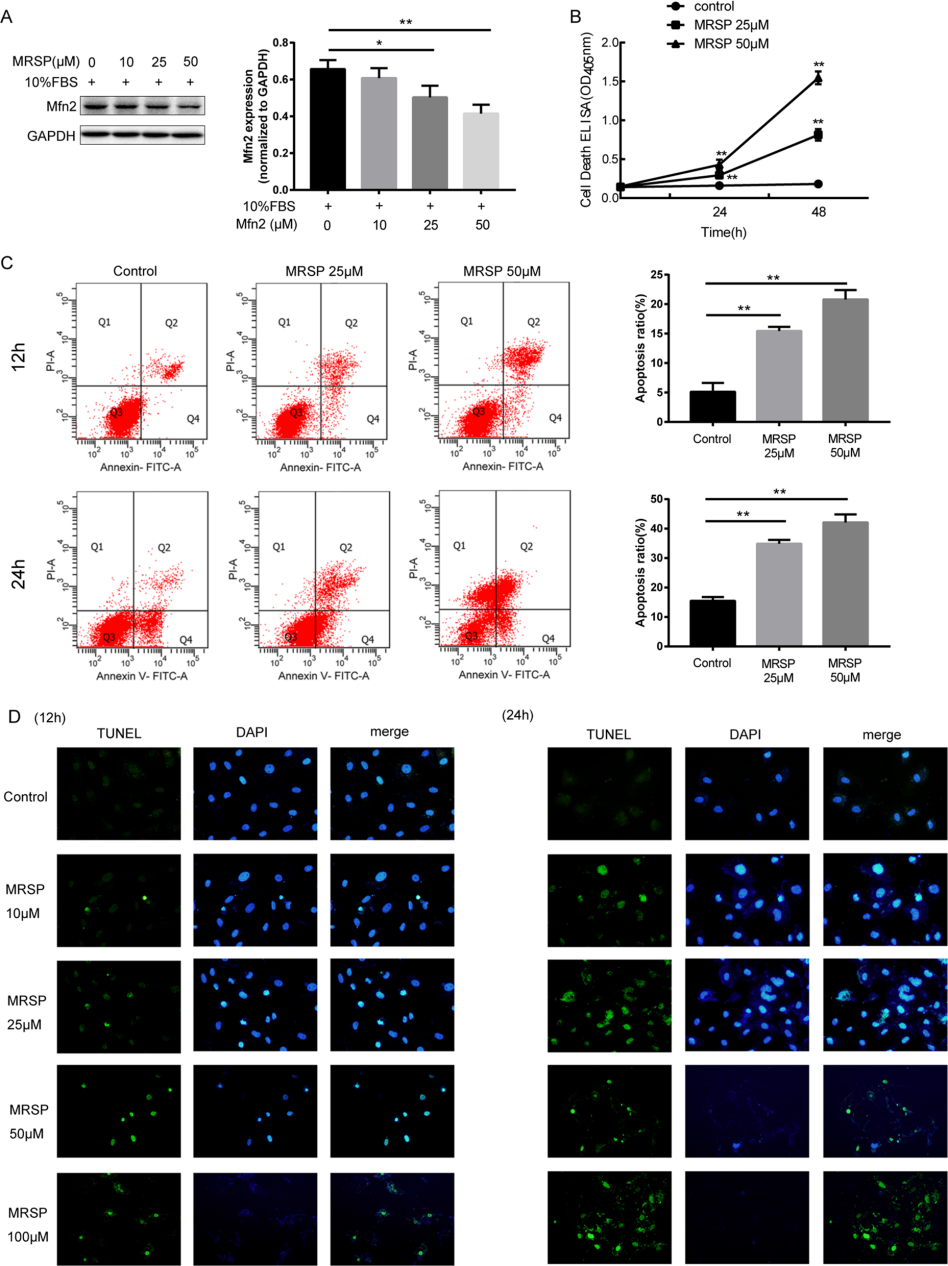
MRSP treatment increased the apoptotic percentage of VSMCs. **A** The level of endogenous Mfn-2 upon treatment with MRSP. **B** The OD_405_ of VSMCs treated with different concentrations of MRSP at 12 h and 24 h using Cell Death ELISAs. **C** Flow cytometry and **D** TUNEL staining were used to analyze MRSP-induced apoptosis of VSMCs in different groups. n = 3 for this experiment. *p < 0.05, **p < 0.01

Then, the effects of MRSP on VSMC apoptosis and the relevant mechanisms were investigated. First, we detect the level of endogenous Mfn-2 and found that MRSP caused a decrease of endogenous Mfn-2 in a dose-dependent manner (Fig. 2A). There was a significant time- and concentration-dependent increase in MRSP-induced VSMC apoptosis, as evidenced by Cell Death ELISA (Fig. 2B) results showing that OD_405_ was significantly increased at 12 h, which lasted up to 24 h (p < 0.01). Moreover, MRSP (25 μM and 50 μM) notably enhanced apoptosis at the 12 h and 24 h time points respectively (p < 0.01) according to flow cytometry data (Fig. 2C). Consistently, TUNEL staining revealed that MRSP treatment induced obvious percentage alterations in the fraction of apoptotic cells (Fig. 2D). The results suggested that MRSP induced effective proapoptotic effects in VSMCs, and DNA condensation and fragmentation and even nuclear disappearance occurred in a time- and concentration-dependent manner after MRSP administration.

### MRSP activated the mitochondrial apoptotic pathway of VSMCs

**Fig. 3:**
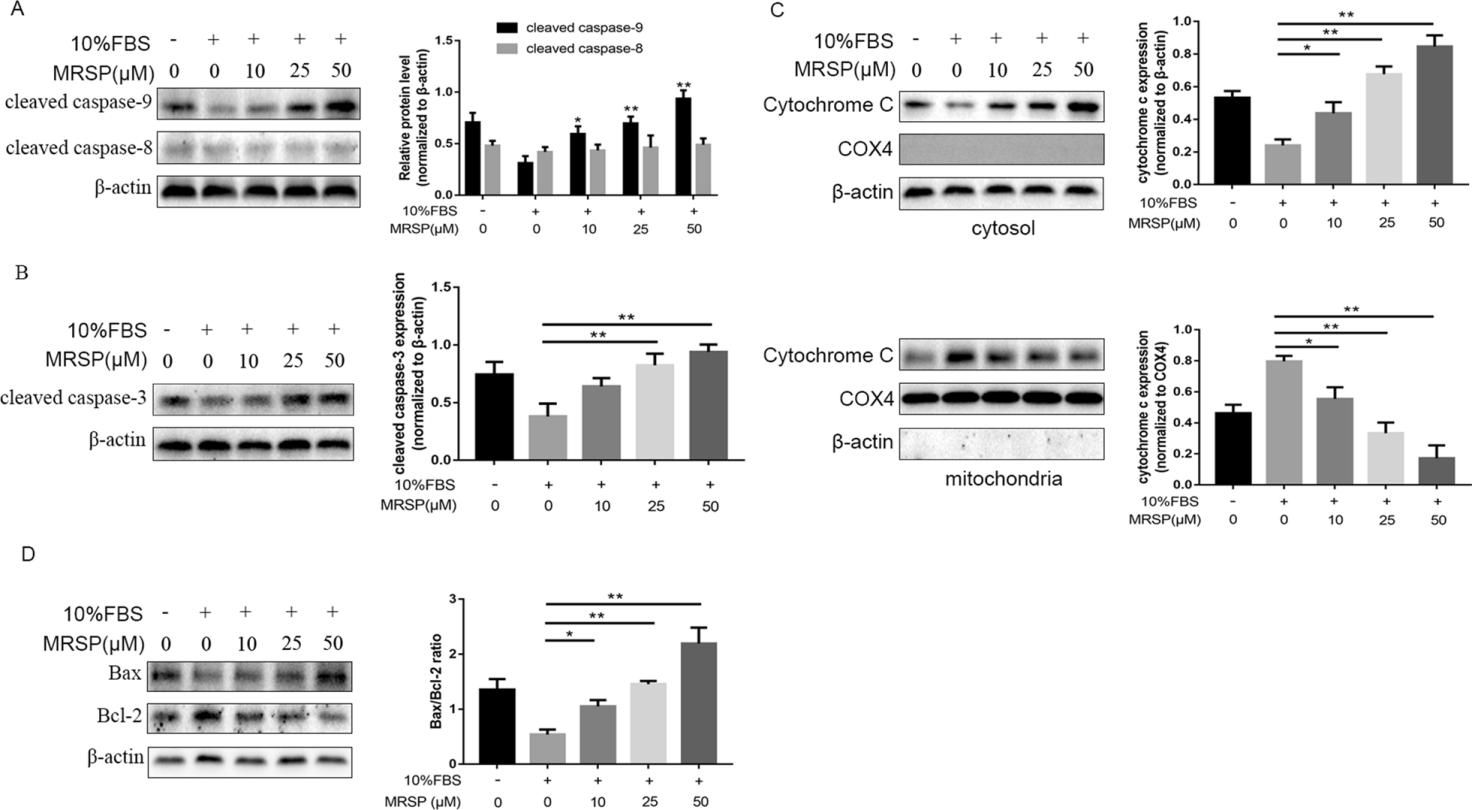
MRSP activated the mitochondrial apoptotic pathway. **A**–**B** The effects of MRSP on the activation of caspase-8, caspase-9 and caspase-3 in different groups. **C** The release of cytochrome c from mitochondria to the cytoplasm after treatment with MRSP was examined by western blots. **D** The ratio of Bax/Bcl-2 induced by MRSP. n = 3 for this experiment. *p < 0.05, **p < 0.01

To further explore the specific mechanisms responsible for MRSP-induced apoptosis, we detected the activation of caspase-8 and caspase-9 in VSMCs. Firstly, we found that there is no difference between the group of HIV1TAT alone and the control group according to the results of Western blotting of caspase-9, caspase-3 and the release of cytochrome c from mitochondria to the cytoplasm (Additional file 1: Fig. S1). So we used "nothing" as control. The MRSP-treated group showed a large increase in the cleavage of procaspase-9 rather than that of procaspase-8 (Fig. 3A). The level of caspase-9 activation was increased in a concentration-dependent manner with MRSP intervention. Likewise, VSMCs treated with MRSP displayed increased activation of caspase-3 with a similar concentration profile (Fig. 3B). These results showed that MRSP promoted VSMC apoptosis through activation of caspase-9 and caspase-3, but not caspase-8, suggesting that the mitochondrial apoptotic pathway was likely responsible for MRSP-induced VSMC apoptosis. This speculation was supported by the fact that MRSP treatment led to a significant increase in the release of cytochrome c from mitochondria to the cytoplasm. The percentage of cytosolic cytochrome c was elevated from 24.04 ± 3.64% (n = 3) to 84.66 ± 6.90% (p < 0.01) in the cells treated with 50 μM MRSP (Fig. 3C).

### MRSP oppositely regulated the levels of mitochondrial Bcl2 and Bax protein abundance

To further explore the signaling events associated with MRSP-induced apoptosis, we assayed the potential effects of MRSP on Bcl-2 family members. Bcl-2, an antiapoptotic member of the family, was markedly reduced in the VSMCs treated with MRSP compared with that of the control group. In contrast, the expression of Bax protein, a proapoptotic protein, was obviously elevated. Thus, MRSP treatment induced an increase in the Bax/Bcl-2 ratio (Fig. 3D), which was responsible for the activation of the mitochondrial apoptotic pathway.

### MRSP induced VSMC apoptosis by inhibiting Akt activation

**Fig. 4:**
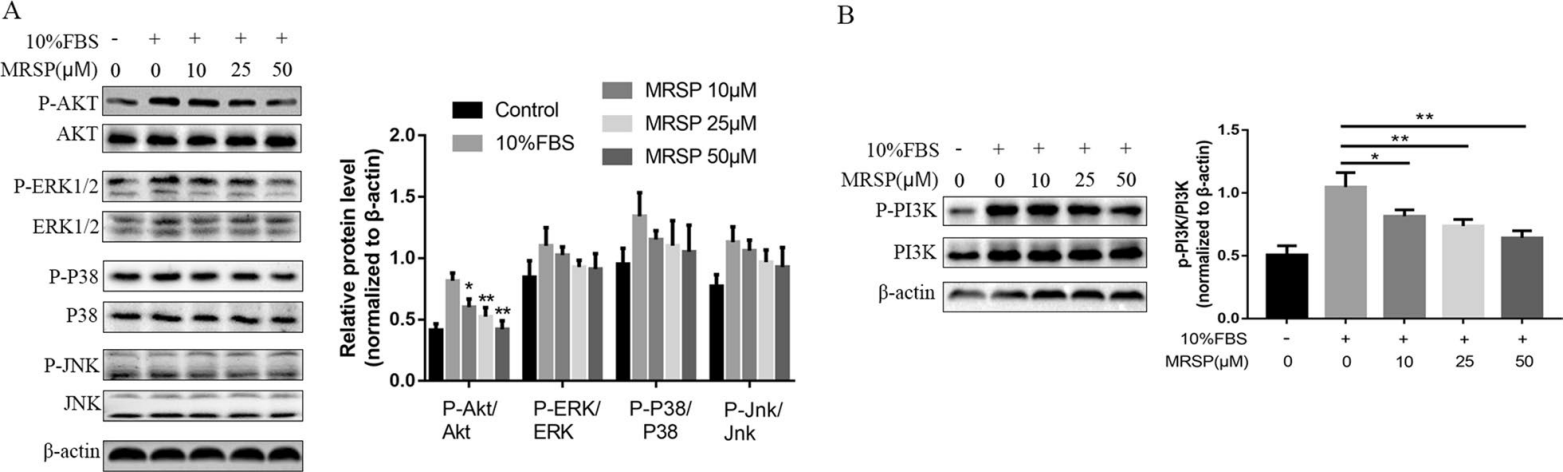
MRSP affected the phosphorylation of molecules involved in Ras signaling pathway. **A** Phosphorylation levels of Akt, ERK, p38 and JNK were measured by western blots. **B** The phosphorylated status of PI3K, an upstream molecule of Akt was examined. n = 3 for this experiment. *p < 0.05, **p < 0.01

Ras mediated cell survival via activation of an array of downstream signaling cascades, including the Ras-Raf-MEK-ERK/MAPK, PI3K-Akt, MEKK3-P38 and MEKK4-JNK signaling pathways. Our results had indicated that MRSP suppressed the activation of the ERK/MAPK pathway, leading to VSMC growth arrest. Then, we further assayed the above pathways in VSMC apoptosis induced by MRSP by testing the phosphorylation status of Akt, ERK1/2, p38 and JNK. Remarkably, MRSP significantly weakened FBS-induced Akt activation in a concentration-dependent manner, as evidenced by suppression of Akt phosphorylation at Ser473 in the cells treated with MRSP. However, under the same experimental conditions, there was a downward trend of ERK1/2 phosphorylation and the phosphorylation of p38 and JNK was not significantly affected (Fig. 4A). Likewise, the level of phosphorylated PI3K, the upstream molecule, was reduced in groups the treated with MRSP (Fig. 4B).

**Fig. 5:**
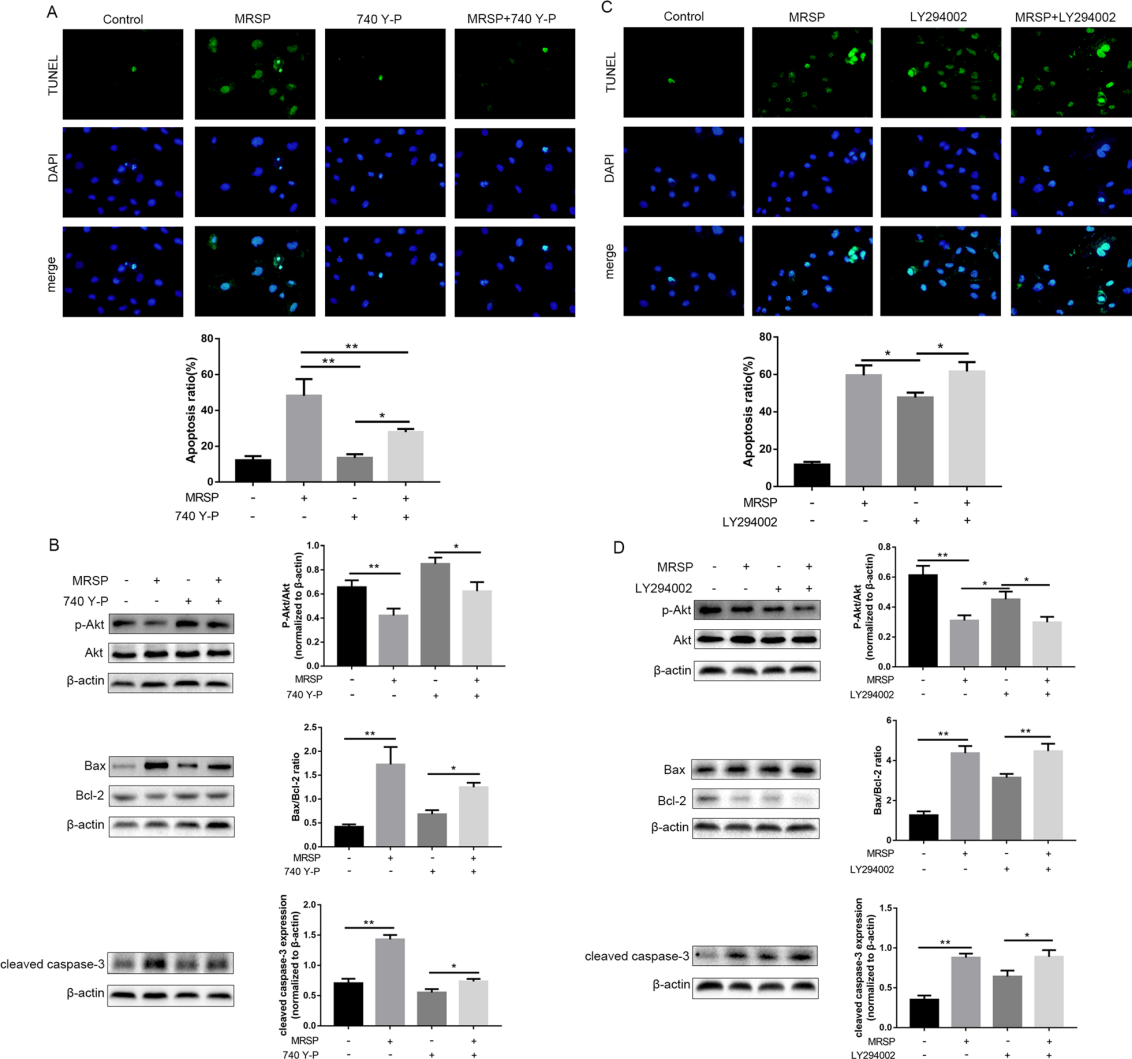
Effects of MRSP on VSMC apoptosis after pretreatment with 740 Y-P or LY294002. **A** The apoptosis induced by MRSP of VSMCs pretreated with 740 Y-P was examined using TUNEL staining. **B** The influence of MRSP on Akt phosphorylation, Bcl-2, Bax expression and caspase-3 activation of VSMCs preincubated with 740 Y-P. **C** The apoptosis induced by MRSP of VSMCs pretreated with LY294002 was examined using TUNEL staining. **D** The influence of MRSP on Akt phosphorylation, Bcl-2, Bax expression and caspase-3 activation of VSMCs incubated with LY294002. n = 3 for this experiment. *p < 0.05, **p < 0.01

Furthermore, we pretreated VSMCs with the PI3K agonist 740 Y-P or MRSP and then treated them with 10% FBS for 12 h. We found that 740 Y-P could reverse the apoptotic effects of MRSP. Indeed, cotreatment of cells with 740 Y-P not only weakened MRSP-mediated inhibition of Akt phosphorylation and upregulation of Bax expression but also prevented caspase-3 activation, downregulation of Bcl-2 expression and VSMC apoptosis, as shown by TUNEL staining (Fig. 5A and B), implying that the inhibition of the PI3K signaling pathway is an important mechanism of MRSP in VSMC apoptosis.

Then, we used LY294002, a specific inhibitor for PI3K, to investigate whether it affects VSMC apoptosis. VSMCs were treated with LY294002 for 12 h; the results showed that LY294002 also induced apoptosis in proliferative cells and that MRSP could increase LY294002 pretreatment-induced apoptosis according to TUNEL staining, and the protein expression of Bax, Bcl-2 and caspase-3 activation (Fig. 5C and D). These data suggested that the apoptotic effect of MRSP, at least partly, was attributed to the PI3K/Akt signaling pathway.

### Inhibitory effects of MRSP on balloon injury-induced neointimal formation

**Fig. 6:**
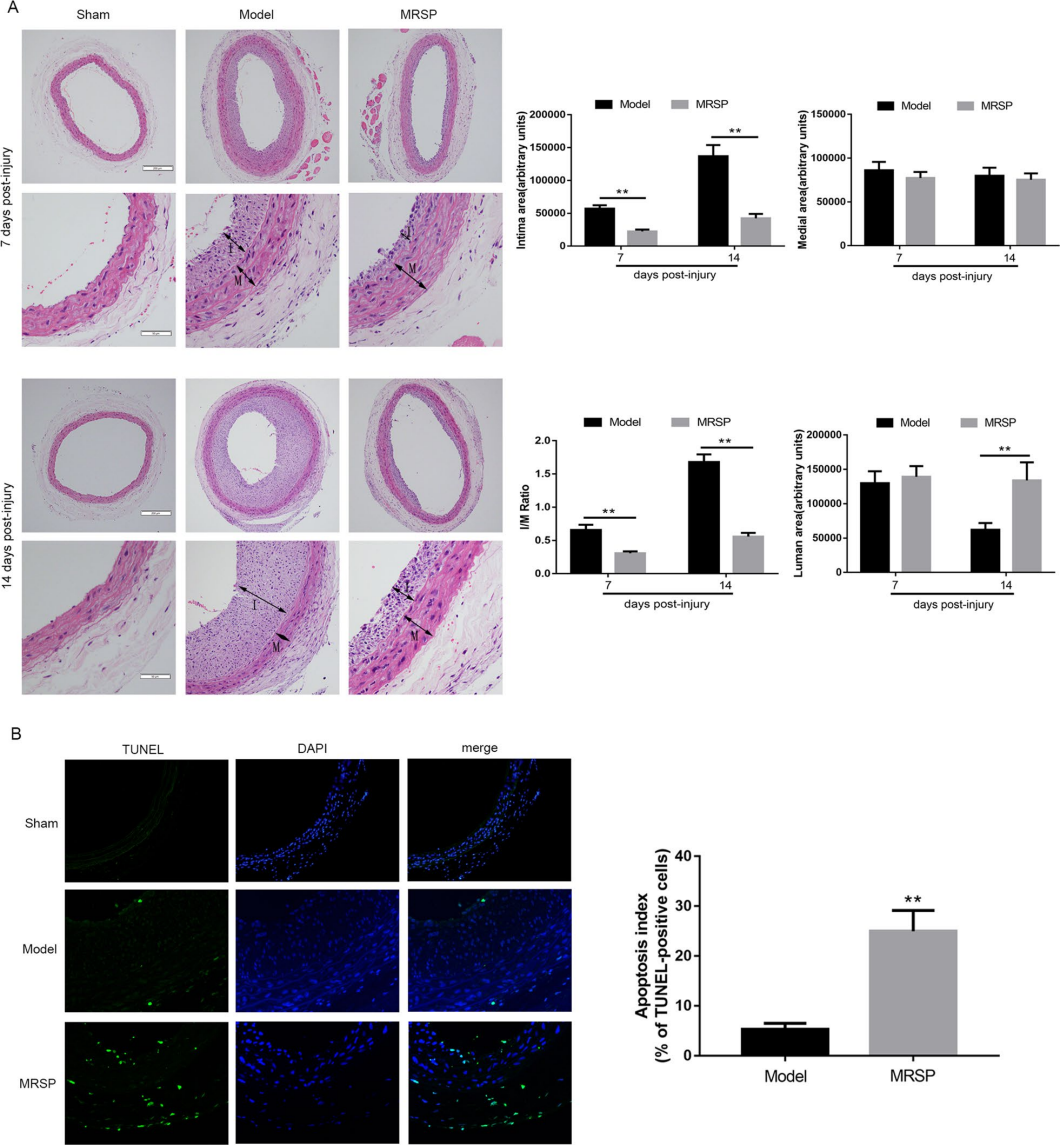
Therapeutic effect of MRSP on apoptosis after balloon injury in vivo. **A** Representative microphotograph of HE staining in different groups at one or two weeks after balloon injury, and analysis of the intima area, media area, I/M ratio and lumen area. **B** Quantification of apoptosis performed at day 7 by TUNEL staining in different groups. n = 6 for each group. *p < 0.05, **p < 0.01 vs. the model group

**Fig. 7:**
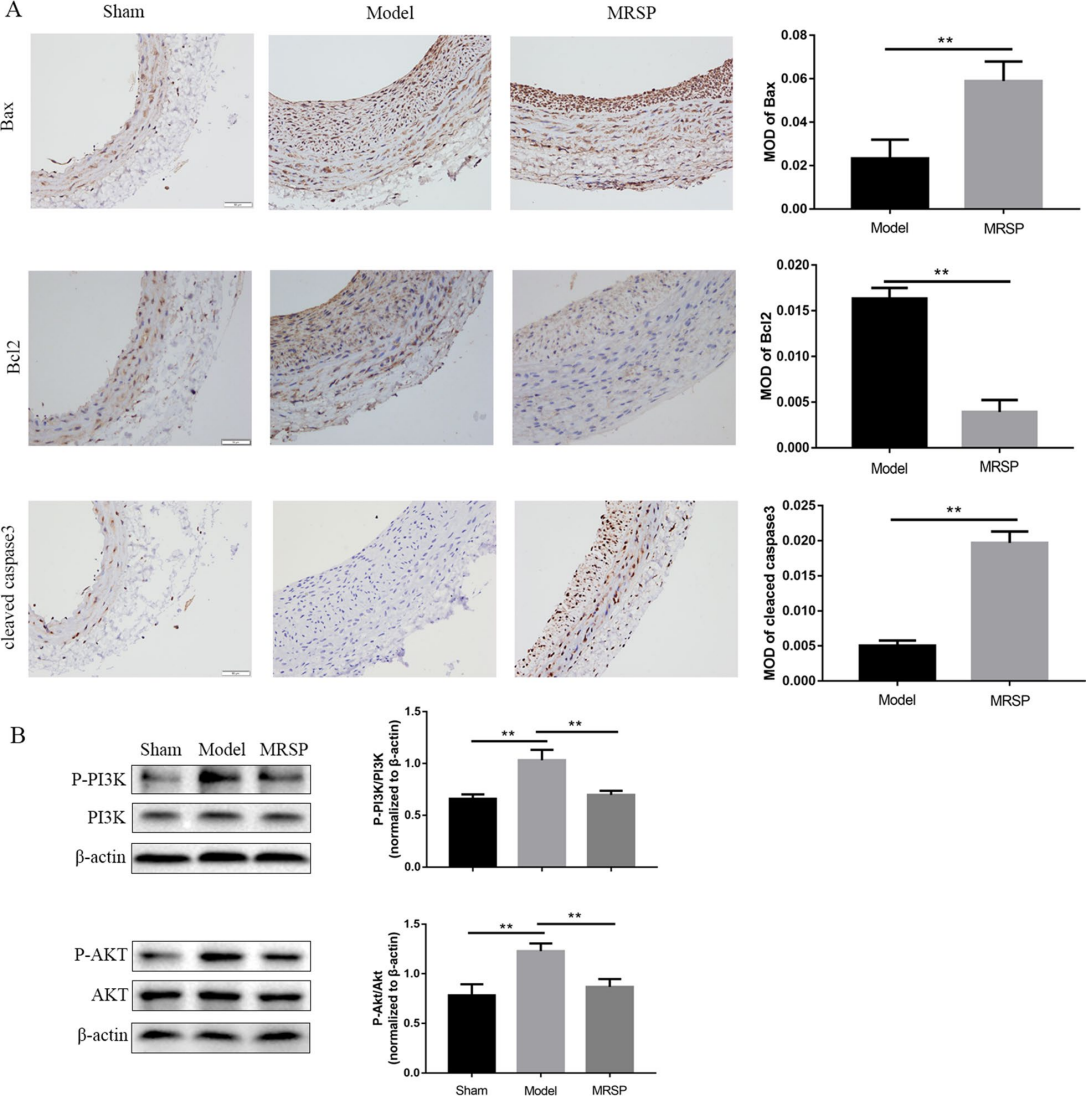
Effects of MRSP on the signaling molecules in different groups. **A** Immunostaining for Bax, Bcl-2 and cleaved caspase-3 in the carotid arteries. **B** Signal transduction of PI3K/Akt pathway was examined. n = 6 for each group. *p < 0.05, **p < 0.01 vs. the model group

To further determine the pathological importance of the proapoptotic effect of MRSP in vivo, rat carotid arteries were subjected to balloon injury as described previously [16] and simultaneously treated with MRSP or nothing. On days 7 and 14 after the operation, rat arteries from different groups were obtained for analysis. Figure 6A shows that representative examples of HE-stained vessels. The balloon injury-induced increase in the ratio of intima to media area was markedly attenuated by MRSP. In addition, the percentage of TUNEL-positive cells was further augmented from 5.33 ± 1.18% in the model group to 24.97 ± 4.16% in the MRSP group (p < 0.01, Fig. 6B). Subsequently, the efficiency of in vivo Bax, Bcl-2 and caspase-3 activity was tested by immunohistochemical staining with relevant antibodies. As shown in Fig. 7A, one week after the surgery, the Bax and cleaved caspase-3 levels were significantly increased in the arteries treated with MRSP relative to those of the model group. In contrast, the balloon injury-induced increase in the Bcl-2 level was markedly reduced by MRSP.

To confirm the role of MRSP in the PI3K/Akt signaling pathway in vivo, we performed western blotting to detect the phosphorylation levels of the above molecules. MRSP significantly suppressed the phosphorylation of PI3K and Akt (Fig. 7B). These in vivo observations demonstrated that MRSP strongly drove cell apoptosis, thus contributing, at least in part to the inhibition of neointima formation after balloon injury.

## Discussion

Vascular injury induced by balloon angioplasty can activate diverse mechanisms in cells, resulting in neointimal thickening. Thus, clinicians need novel strategies to further reduce the occurrence of this event and achieve better prognostic outcomes. The major finding of the present study was that MRSP, an artificially synthesized peptide, markedly triggered apoptosis in cultured VSMCs and controlled angioplasty-induced neointimal thickening, which was mainly achieved by activating of the mitochondrial apoptotic signaling cascade regulated by the PI3K/Akt survival pathway, providing insights into the development of anti-RS therapy using MRSP. Taken together, our study provides data for the potential application of MRSP in a drug-eluting stent to reduce neointimal hyperplasia after angioplasty.

Our data identified MRSP as a potential novel therapeutic regimen for RS. Drug-eluting stents using paclitaxel and rapamycin that effectively inhibit proliferation of VSMCs have markedly lowered the incidence of stenosis [17]. However, there remains residual risk and new strategies involving new mechanisms are warranted. In the process of RS, the imbalance of excessive proliferation and insufficient apoptosis might cause abnormal accumulation of cells in the intima, which contributes to atherosclerosis or RS [13, 18]. Kamenz et al. showed that a lower incidence in cellular apoptosis—not exceeding 1% of the total neointimal cell number—in comparison to increased cellular proliferation potentially explained the increase in cell number in the intima [18]. Homeostasis between cell apoptosis and proliferation was achieved in restenotic lesions until day 28 after vascular injury in an animal model. Interestingly, approximately 40% of cells underwent apoptosis day 9 post injury in the thickening intima and decreased to 10% at 4 weeks [19]. Isner et al. reported that the occurrence of apoptosis was greater in restenotic lesions than in primary atheromatous plaques, especially in those with high proliferative activity, implying that the increase in apoptosis during RS may be a compensatory mechanism for the excessive proliferation of cells [20]. Thus, targeting early apoptosis represents an attractive approach. We revealed that MRSP effectively suppressed neointimal hyperplasia likely by promoting apoptosis of VSMCs in a classic rat model of vascular injury. The observations in rats were further elaborated by the in vitro data showing that MRSP is cellular internalized from the extracellular fluid and causes apoptosis of VSMCs. Therefore, MRSP targeting of VSMC apoptosis might be a feasible strategy to further combat RS.

Fluorescence colocalization analysis demonstrated that MRSP was internalized and probably colocalized with MitoTracker at early time points and trended to transfer to the cell nucleus. However, the nature of MRSP and the detailed intracellular route remain to be elucidated. Mechanistically, there are two independent pathways associated with cellular apoptosis: the mitochondria-cytochrome c pathway and the death receptor-regulated pathway [21–23]. Activation of caspase-8 and caspase-9 are pivotal events in the two pathways [24]. In this study, a significant elevation of cleaved caspase-9 with unaltered cleaved caspase-8 was observed in the MRSP group, which suggested that the proapoptotic behavior of MRSP was likely attributed to the mitochondrial death pathway. The antiapoptotic and proapoptotic members of the Bcl-2 family are closely related to mitochondrial apoptotic pathway [25]. The interaction of Bcl-2 and Bax is a determinant of cellular death or survival and the Bax/Bcl-2 ratio is a crucial indicator of VSMC apoptosis [26, 27]. Bax can combine with the other proapoptotic Bcl-2 family members on the outer mitochondrial membrane, leading to a change in its permeability and the release of cytochrome c, subsequently activating caspase-9 and caspase-3, and further causing cell apoptosis [25, 28, 29]. Bcl-2, the other member of the Bcl2 family, exerted an influence on promoting cellular survival and suppressing the actions of pro-apoptotic proteins [30]. Here, we demonstrated that MRSP increased the ratio of Bax/Bcl-2, further increasing the release of cytochrome c from the mitochondria to the cytoplasm, and activating caspase-9 and caspase-3 but not caspase-8, resulting in cell apoptosis in vitro. In vivo data showed that MRSP significantly enhanced the apoptosis of intima cells and further weakened neointimal hyperplasia after carotid artery balloon injury in rats. In further studies, we will use specific blockers of different apoptotic pathways to verify this conclusion.

**Fig. 8:**
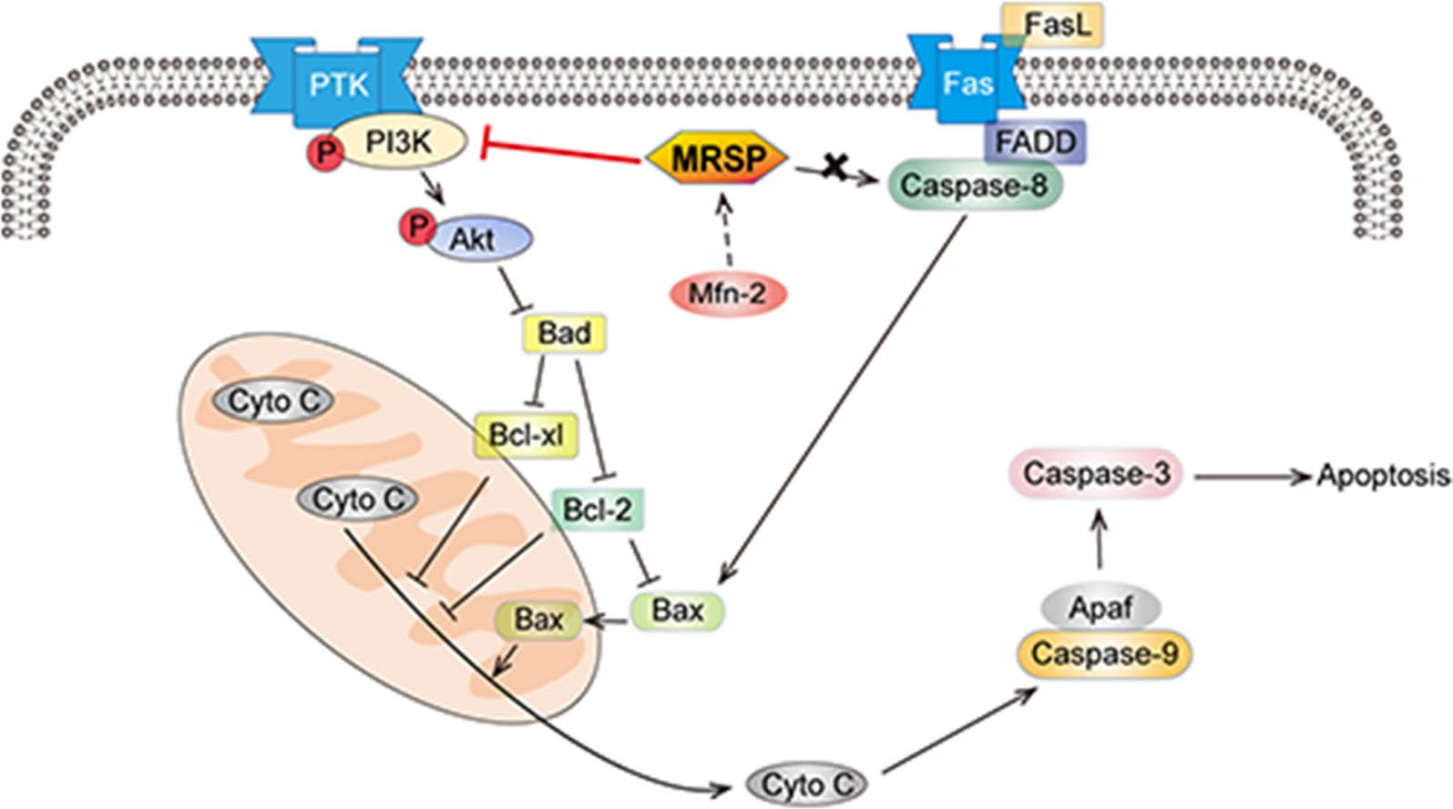
Schematic description of the protective mechanisms by which MRSP promoted the apoptosis in VSMCs and the vascular wall after balloon injury. Cyto C, cytochrome c

Moreover, the PI3K/Akt pathway mediated the apoptotic effects of MRSP on VSMCs as well as on neointimal hyperplasia in rats. In the present study, MRSP caused dose-dependent downregulation of the phosphorylated levels of PI3K and Akt, which ultimately resulted in increased apoptosis of VSMCs. Akt exerts an important effect in the pathogenesis of cardiovascular diseases and is known to mediate cell migration and survival [31, 32]. The PI3K/Akt signaling pathway is a vital mediator of apoptosis attributed to certain downstream target molecules including Bad, Bax, caspase-9, NF-κB and p21, which are closely related to apoptosis [25, 33, 34]. Activation of the PI3K/Akt pathway is a contributor to in-stent RS and the Akt1 siRNA nanoparticle eluting stent was shown to suppress postangioplasty RS [35, 36]. These effects identify the PI3K/Akt pathway as a therapeutic target in treating cardiovascular proliferative disorders. In the present work, the decrease in Akt phosphorylation was paralleled by activation of the mitochondrial apoptotic signaling cascade. These data indicated that MRSP can promote apoptosis, potentially due to MRSP-mediated inhibition of the PI3K/Akt signaling pathway (Fig. 8). This speculation was proven by the evidence that MRSP obviously blunted the activation of PI3K and Akt but not ERK1/2, JNK, or P38, implying that MRSP activated the PI3K/Akt signaling pathway independent of ERK1/2, JNK and p38 signaling. To further understand the role of MRSP-induced PI3K/Akt signaling in the process of VSMC apoptosis, we analyzed the effect of PI3K/Akt agonists and inhibitors on VSMC apoptosis. As expected, the data showed that the PI3K agonist diminished the MRSP-induced apoptotic effect, whereas the inhibitor strengthened the influence. Notably, PI3K/Akt pathway was strongly associated with tumor progression, indicating that MRSP might also have good potential in antitumor therapy. A related work is in progress in our laboratories and might promote the application of the same mechanism in treating RS.

## Conclusion

Taken together, the present study provided the first evidence that MRSP treatment results in a strong proapoptotic effect in VSMCs and this effect was achieved by activating the mitochondrial apoptotic signaling cascade through downregulation of the PI3K/Akt pathway, which might be the key potential mechanism of the anti-RS effects. This experiment confirmed that MRSP could attenuate intimal hyperplasia after balloon injury, providing an experimental basis for further clinical application of MRSP such as stent coating drug treatment of RS.

## Supplementary Information

**Additional file 1: Fig. S1.** The difference between the group of HIV1TAT alone and the control group. (A-B) The effects on the activation of caspase-9 and caspase-3 in different groups. (C) The release of cytochrome c from mitochondria to the cytoplasm was examined by western blots. (D) The ratio of Bax/Bcl-2. n = 3 for this experiment.

## Data Availability

The data that support the findings of this study are available from the corresponding author upon reasonable request.

## Abbreviations

Mfn-2: Mitofusion 2
MRSP: Mfn-2 related synthetic peptide
VSMCs: Vascular smooth muscle cells
RS: Postoperative restenosis
DMEM: Dulbecco's modified Eagle medium
FBS: Fetal bovine serum
COX4: Cytochrome c oxidase 4
BCA: Bicinchoninic acid
PVDF: Polyvinylidene difluoride
HE: Hematoxylin and eosin
I/M: Intima-to-media
ANOVA: One-way analysis of variance
