# Supplementary figures and images for: A new Mfn-2 related synthetic peptide promotes vascular smooth muscle cell apoptosis via regulating the mitochondrial apoptotic pathway by inhibiting Akt signaling
Source: J Transl Med. 2021 Sep 19;19:395. doi: 10.1186/s12967-021-03064-1 (PMC8451139; doi:10.1186/s12967-021-03064-1)

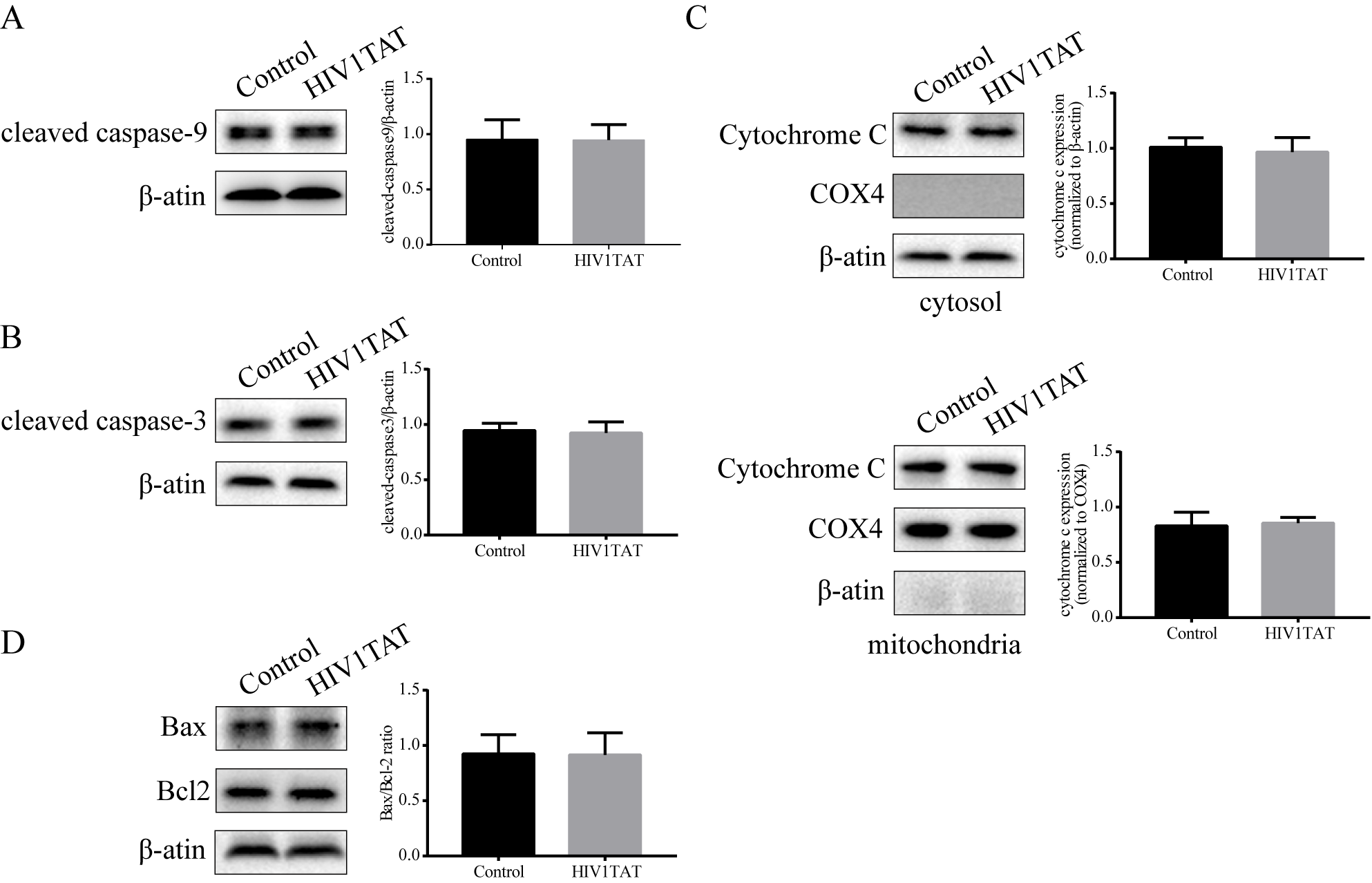

Supplement: Supplementary file 1 — Additional file 1: Fig. S1. The difference between the group of HIV1TAT alone and the control group. (A-B) The effects on the activation of caspase-9 and caspase-3 in different groups. (C) The release of cytochrome c from mitochondria to the cytoplasm was examined by western blots. (D) The ratio of Bax/Bcl-2. n = 3 for this experiment. [file 12967_2021_3064_MOESM1_ESM.tif]
